# Formation of size-dependent and conductive phase on lithium iron phosphate during carbon coating

**DOI:** 10.1038/s41467-018-03324-7

**Published:** 2018-03-02

**Authors:** Yulong Liu, Jian Liu, Jiajun Wang, Mohammad Norouzi Banis, Biwei Xiao, Andrew Lushington, Wei Xiao, Ruying Li, Tsun-Kong Sham, Guoxian Liang, Xueliang Sun

**Affiliations:** 1 Department of Mechanical and Materials Engineering, Western University, London, ON N6A 5B9 Canada; 2 Department of Chemistry, Western University, London, ON N6A 5B7 Canada; 3 Johnson Matthey, 280 Ave. Liberté, Candiac, QC J5R 6X1 Canada

## Abstract

Carbon coating is a commonly employed technique for improving the conductivity of active materials in lithium ion batteries. The carbon coating process involves pyrolysis of organic substance on lithium iron phosphate particles at elevated temperature to create a highly reducing atmosphere. This may trigger the formation of secondary phases in the active materials. Here, we observe a conductive phase during the carbon coating process of lithium iron phosphate and the phase content is size, temperature, and annealing atmosphere dependent. The formation of this phase is related to the reducing capability of the carbon coating process. This finding can guide us to control the phase composition of carbon-coated lithium iron phosphate and to tune its quality during the manufacturing process.

## Abstract (web summary)

Coating the active materials of interest with carbon is a widely employed way to boost the performance of lithium ion batteries. Here the authors show the formation of a conductive phase on LiFePO_4_ during carbon coating, which is size, annealing temperature and reduction atmosphere dependent.

## Introduction

Since the first report in 1997, olivine LiFePO_4_ (LFP) as an environmentally benign and a safer cathode material has been widely studied in the field of energy storage^1^. Considerable efforts have been devoted toward improving the intrinsic low electronic and ionic conductivity of this material^2–4^. Surface carbon coating is often used to increase the electrical conductivity of LFP and has proven to be an effective strategy^4,5^. This has led to significant progress for the wide-spread application of LFP in commercial lithium ion batteries during the last two decades^6–8^. However, an in-depth understanding of surface chemistry changes during the carbon coating process remains elusive. Carbon coating usually involves a strong reducing environment and often requires high temperature. This increases the reaction kinetics between the surface of LFP and the supplied carbon^9^. As a result, secondary phases will form on the surface, thereby altering the electronic/ionic conductivity of LFP^10–14^. Previously, a Fe_2_P_2_O_7_ secondary phase was observed on the surface of hydrothermally synthesized LFP. The origin of such impurity phase formation is ascribed to more Fe_Li_ antisite formed at lower temperature synthesis than high temperature solid-state reactions^15–17^.  The formation of another secondary phase, iron phosphide, has been shown to be closely related to the annealing procedure employed and undoubtly influence the physical properies of LFP. Annealing the LFP at different temperatures/atmosphere, iron phosphides (FeP, Fe_2_P, and Fe_3_P) were detected with XRD^18,19^. By controlling the content of the iron phosphides, the long-term cycle performance and rate performance of carbon-coated LFP was improved^20–22^. In addition, work on the carbon quality of LFP suggested that more graphitic carbon layers were easily formed with iron phosphide inside LFP^9^. Because the carbon coating process on LFP is a multi-component system at small scale, it is very difficult to define the presence of secondary phase, to map the distribution of secondary phase, to record the change of secondary phase, and the control of secondary phase. Therefore, understanding the formation mechanism for emergence of secondary phases would be highly beneficial, especially for LFP manufacturers.

In our previous study, we found interface reactions occurred between the carbon layer and LFP during the carbon coating process. The evaporation of lithium at high temperature results in the formation of an inert impurity phase Fe_2_P_2_O_7_. With the help of advanced techniques, we clearly recorded the location and distribution of surface phases and hypothesized the formation of Fe_2_P_2_O_7_^23^. Our next study shows that the amount of the Fe_2_P_2_O_7_ phase can be controlled, and even be fully removed by tuning parameters applied during the carbon coating process. However, removal of a negative secondary phase in LFP is not the only objective to produce high quality LFP. It would also be essential to improve the electrochemical performance of LFP by tuning electronic conductivity. There have been numerous attempts to increase the electronic conductivity of LFP, such as forming a percolating carbon network on the surface of LFP^12–14^. Furthermore, coating formed on the LFP surface to enhance the lithium ion conductivity, by forming off-stoichiometry composition, is of great interest. Surface engineering on the LFP with the aim of improving the electrochemical performance has been a very promising direction in this field^14,24–26^. Our research goal is to engineer the chemical composition of LFP surface via carbon coating. This method may be effective in creating an appropriate off-stoichiometry LFP with uniform carbon coating on the surface. Such a unique carbon-coated LFP materials could simultaneously resolve both low lithium ion conductivity and electronic conductivity. Due to the highly complex nature of interface reaction, there is no direct way to visualize and monitor the surface chemistry change during carbon coating process. Recently, melt-casting has been shown to be a promising technique in preparing LFP^27,28^. After polishing the surface of melt-casted LFP ingot, a large flat surface can be obtained. A mirror surface takes the advantage of visualization of detailed surface chemistry changes during carbon coating process, which would greatly help us to understand the reaction at the interface^9^.

Here in this work, LFP ingot and LFP particles, with different sizes, have been used to demonstrate and present a size-dependent conductive secondary phase formation during carbon coating. Furthermore, surface phase changes are correlated to temperature and reducing atmosphere. Therefore, formation of secondary phase is controlled by LFP particle size, annealing temperature, and reducing atmosphere, simultaneously. LFP particles with appropriate amount of conductive phase are obtained by carefully tuning coating parameters during the carbon coating process. The improved electrochemical performance of LFP materials with conductive phase suggests that such secondary phase has a positive influence. From a thermodynamic point of view, we propose a unified mechanism that can be used to describe the formation of Fe_2_P phase under different circumstances. In particular, we use LFP ingot sample to demonstrate the direct visualization of this phase change mechanism during carbon coating. Our study on carbon-coated LFP may also enlighten the interests on the interface chemistry research, especially, reactions of insulating materials in reducing atmosphere.

## Results

### Surface phase formation

**Fig. 1:**
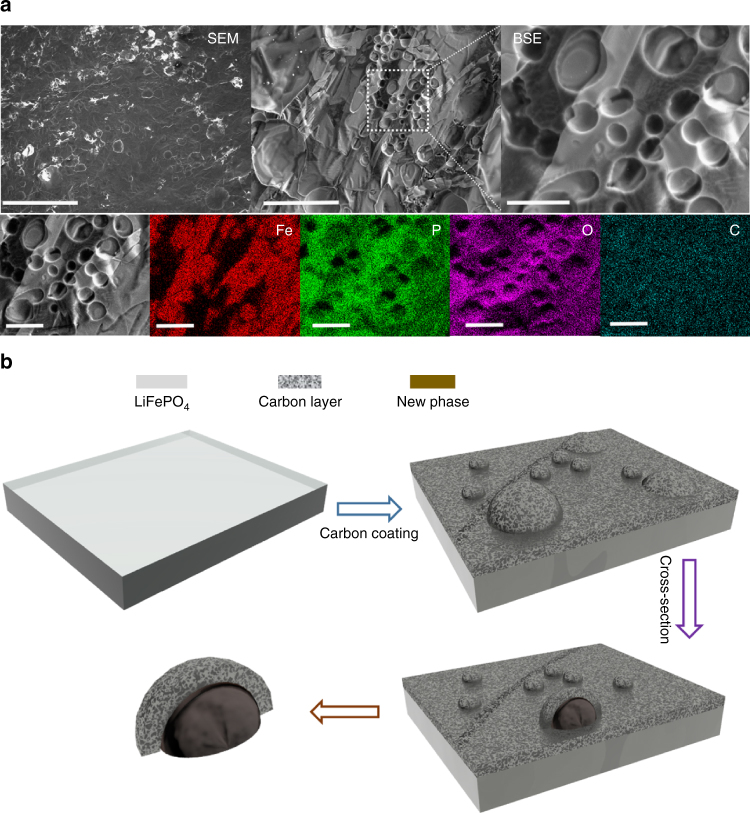
Surface conductive phase formation during carbon coating process. **a** Upper panel: SEM images (left side), BSE images (middle and right); lower panel: EDS mapping of surface conductive phase formation on LiFePO_4_. **b** Schematic representation of new phase formation on LiFePO_4_. Scale bar, 1 mm (SEM image), 200 µm (BSE), and 50 µm (right side BSE and lower panel EDS images)

As shown in Fig. 1, an intriguing phenomenon of spherical-like phase growth can be seen on the surface of the carbon-coated LFP ingot at 900 °C, and is distinctively different in morphology from the underlying matrix . A close-up view shows that the spherical-like phase is not uniform, with different sizes and shapes observed at various locations. Using energy-dispersive X-ray spectroscopy (EDS) mapping, this surface phase was assigned as an Fe-rich phase covered by a layer of carbon. In addition, a region enriched in phosphorus and oxygen suggests the presence of lithium phosphates. We propose that these surface changes are correlated to the carbon coating process as these observations are not found under H_2_ reduction or pure Ar atmospheres, where no carbon is presented (Supplementary Fig. 1).

### Characterization of the size-dependent surface phase

**Fig. 2:**
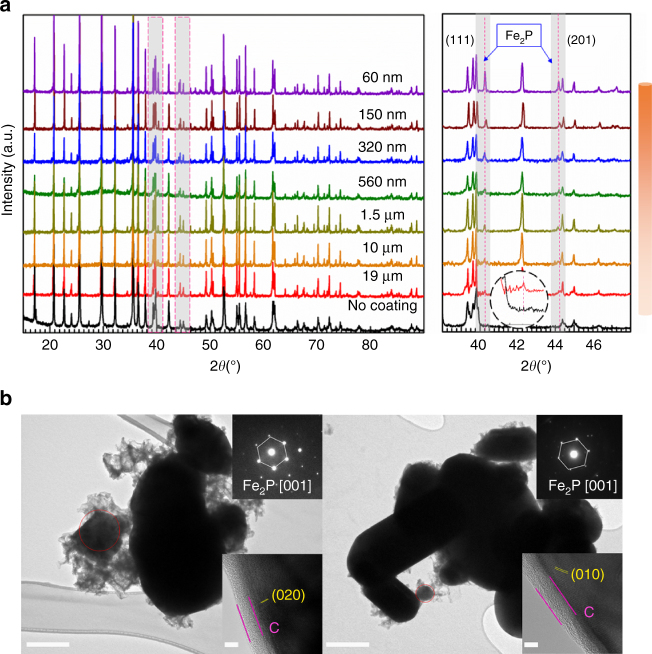
Size-dependent properties of Fe_2_P phase formation. **a** XRD pattern of different size LFP particles after carbon coating at 900 °C. **b** HRTEM Fe_2_P phase for 60 nm LFP and 560 nm LFP annealed at 900 °C. Inset shows the SAED pattern of Fe_2_P phase and HRTEM images of Fe_2_P phase. Scale bar, 500 nm in TEM, 5 nm in HRTEM

To verify our findings, we further extended our study to powder samples with varying sizes. LFP particles ranging from micro-sized (19 µm) to nano-sized (60 nm) were obtained by ball milling LFP ingot sample (size distribution can be found in Supplementary Fig. 2). Figure 2a reveals XRD patterns of carbon-coated LFP with different sizes. Small peaks in the range of 38–48° (gray area) are clearly observed for all LFP particles, and can be assigned to Fe_2_P (JCPDS No 85-1725). As LFP particle size decreases, peak intensities for Fe_2_P intensify (See the inset in Fig. 2a). To support the evidence for a size-dependent relationship on the formation of Fe_2_P, two types of LFP samples with size of 560 nm and 60 nm were characterized using transmission electron microscope (TEM) following carbon coating at 900 °C. It is seen that the size distributions of 560 nm and 60 nm LFP particles has grown due to high temperature (Supplementary Fig. 3). Interestingly, Fig. 2b displays small spherical particles adjacent to larger LFP particles. Selected Area Electron Diffraction (SAED) pattern of that particulate are consistent with Fe_2_P [001]. Moreover, the amount of Fe_2_P phase formed on the 60 nm LFP is bigger than that found for 560 nm LFP. This provides good evidence for a correlation existing between Fe_2_P phase formation and LFP particle size. From the inset of the HRTEM images in Supplementary Fig. 4, the carbon coating on Fe_2_P is about 6 nm and is thicker than the carbon coating on LFP, which is around 3 nm. The smaller size Fe_2_P with thicker carbon coating would be beneficial in improving LFP conductivity.

To get a clear evidence of phase change process, carbon coating was directly observed on powder LFP (nano size LFP) by in-situ SEM observations combined with injection of carbon precursor^23^. Following carbon precursor injection, the particles begin to aggregate and then shrink, ending with a melted product. Using a higher magnification SEM (Supplementary Fig. 5), the formation of secondary phase on the surface of primary LFP particles can be seen.

### Temperature and atmospheric dependent phenomenon

**Fig. 3:**
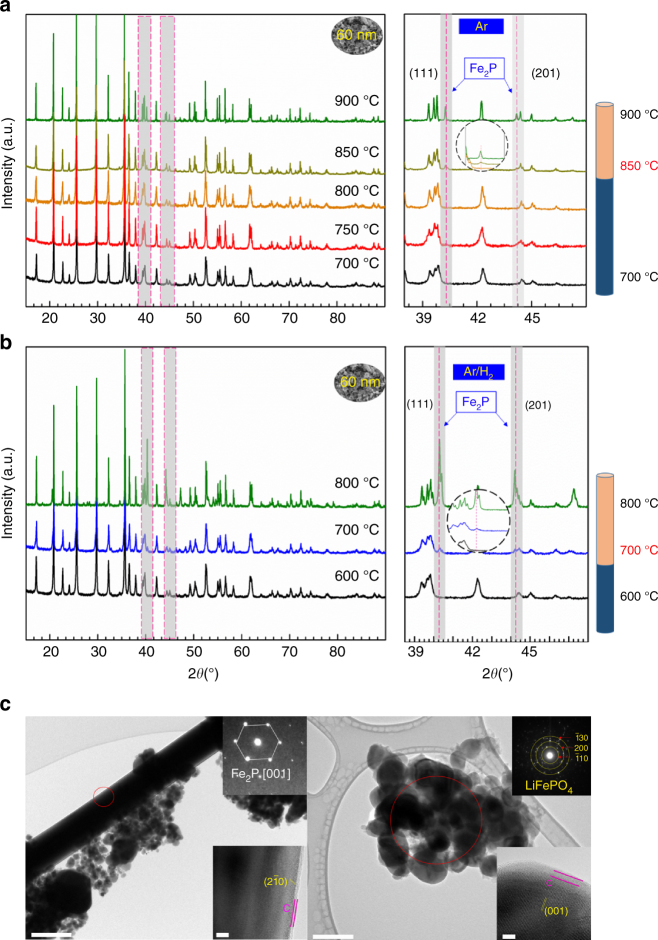
Temperature dependent and atmosphere dependent properties of Fe_2_P phase formation. **a** XRD pattern of 60 nm LFP after carbon coating in Ar from 700–900 °C. **b** XRD pattern of 60 nm LFP after carbon coating in Ar/H_2_ from 600–800 °C. **c** TEM characterization of Fe_2_P phase and LFP for 60 nm LFP annealed in Ar/H_2_ (left side) and Ar (right side) gas at 700 °C. Inset of left picture is the diffraction pattern of Fe_2_P phase and HRTEM images, inset of right picture is the diffraction pattern of LFP phase and HRTEM images. Scale bar, 500 nm in TEM, 5 nm in HRTEM in the left picture. Scale bar, 200 nm in TEM, 5 nm in HRTEM in the right picture

The Fe_2_P new phase could be easily formed at high temperature carbon coating on LFP particles. Figure 3a displays the formation of a new phase following carbon coating of 60 nm LFP at different temperatures. The XRD pattern reveals the formation of Fe_2_P begins at 850 °C and increases in peaks intensity at 900 °C, suggesting that phase formation of Fe_2_P is temperature dependent. If we extend the LFP particle size to 560 nm and 19 µm, 560 nm LFP shows size-dependent phenomenon as 60 nm LFP (Supplementary Fig. 4), but the Fe_2_P phase formation is delayed to 900 °C (Supplementary Fig. 5). This suggests that the formation of Fe_2_P phase is related to both size and temperature.

Apart from temperature, annealing atmosphere is another critical factor during carbon coating of LFP. To investigate the influence of annealing atmosphere, we intentionally introduced H_2_ gas during the carbon coating process and observed the corresponding surface phase changes. Figure 3b displays the atmospheric dependent nature of Fe_2_P impurity phase formation. Carbon coating is performed on 60 nm LFP in the temperature range of 600–800 °C for Ar/H_2_. XRD patterns demonstrate that using an Ar/H_2_ reducing atmosphere, a critical temperature of 700 °C is required for the formation of Fe_2_P phase rather than the 850 °C that is required in a pure Ar atmosphere. For 560 nm LFP, Fe_2_P phase formation temperature reduced to 700 °C, as well as in Ar/H_2_ (Supplementary Fig. 6). Even for 19 µm LFP particles, Fe_2_P phase formation temperature was found to occur at 800 °C, a lower temperature than in pure Ar (900 °C) (Supplementary Fig. 7).

To further identify the Fe_2_P phase distribution in LFP particles, surface morphology was examined following carbon coating at 700 °C. SEM images, shown in Supplementary Fig. 8, demonstrate the formation of large crystals on 60 nm LFP following carbon coating in a more reducing environment (Ar/H_2_). In contrast, no such particles are observed for 60 nm LFP coated in Ar gas. To obtain detailed information about the particles, we picked 60 nm LFP in Ar and Ar/H_2_ for TEM characterization. A large rod-like crystal can be seen for 60 nm-coated LFP in a reducing atmosphere. Based on the SAED diffraction pattern, this feature can be assigned to hexagonal Fe_2_P phase (As shown in Fig. 3c). Nevertheless, no such crystal formation was observed for 60 nm LFP coated in Ar gas, and is further supported by clear polycrystalline LFP diffraction rings. From the HRTEM images in Fig. 3c, the rod-like Fe_2_P phase is shown to be covered by a 2 nm-thick carbon coating, while the nano-LFP is covered with thicker carbon coating (5 nm). Thus, larger Fe_2_P phase with thin carbon coating is observed for 60 nm LFP after carbon coating in Ar/H_2_.

### Electrochemical performance of LFP with new phase

**Fig. 4:**
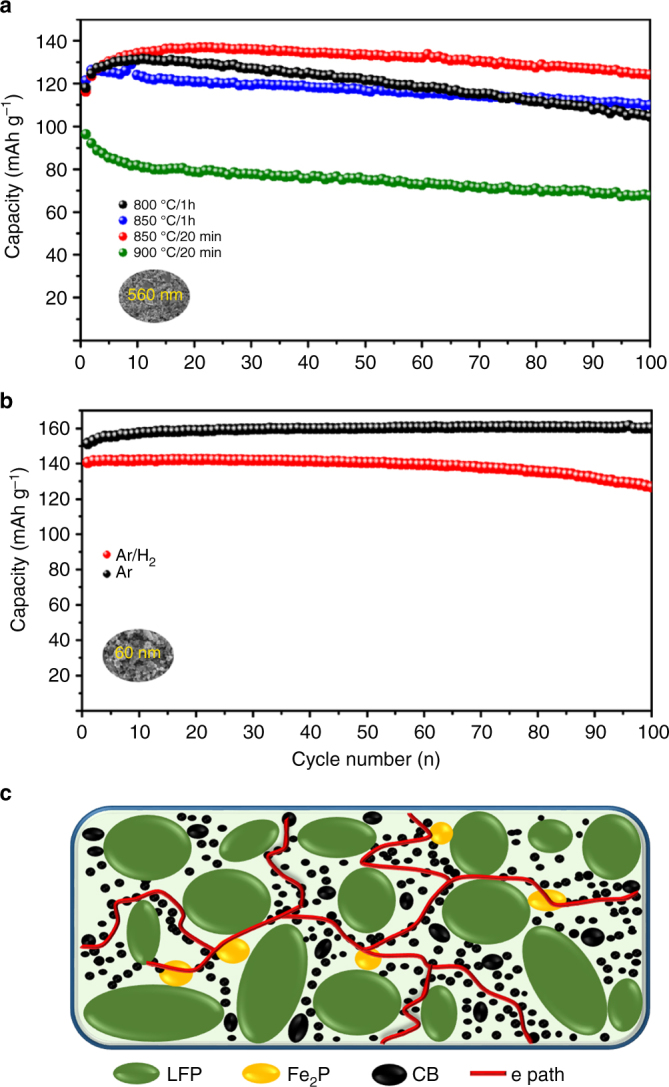
Positive effect of conductive Fe_2_P phase in LFP. **a** Electrochemical properties of 560 nm LFP with different amount of Fe_2_P. **b** 60 nm LFP annealed in different atmosphere. **c** Schematic diagram show the positive effect of Fe_2_P with percolation conduction network

In order to verify the electronic conductivity improvement brought on by Fe_2_P, electrochemical performance of LFP was evaluated. As discussed above, Fe_2_P phase formation in a reducing environment results in the formation of large crystals covered by a thin coating of carbon. This feature may hinder lithium ion transport. Furthermore, SEM and TEM images of 60 nm LFP coated at 900 °C in Ar exhibits large Fe_2_P and LFP particles agglomerated together due to the elevated temperature employed (Supplementary Figs. 4 and 5). Finally, we chose 560 nm LFP to illustrate the effect Fe_2_P on the electrochemical performance of LFP. Figure 4a displays the electrochemical performance of 560 nm LFP particles, following carbon coating under various conditions. Clearly, 560 nm LFP annealed at 900 °C has decreased capacity and cycle performance, compared to pure LFP samples, demonstrating only 67 mAh g^–1^ of capacity after 100 cycles. Two possible reasons may be given for the decreased capacity observed. The first is related to the agglomeration of LFP particles at high temperature, as seen in Supplementary Fig. 4. The second reason is the high content of Fe_2_P phase resulting in decreased lithium conductivity. To verify the influence of Fe_2_P phase content on performance, two 560 nm LFP samples were prepared by annealing them at 850 °C for 20 min and 1 h, respectively. The LFP sample annealed at 850 °C presents less Fe_2_P phase in LFP than 900 °C, as indicated by the XRD pattern in Supplementary Fig. 6, while the sample coated at 800 °C is pure LFP phase. However, the electrochemical performance of the sample annealed at 850 °C and 800 °C demonstrates that the one annealed at a higher temperature results in improved performance. Although the 800 °C-coated LFP sample exhibits a high capacity in the first 60 cycles, the capacity quickly fades. Conversely, after 100 cycles, the 850 °C-coated LFP sample manages to deliver 109 mAh g^–1^, while the 800 °C-coated LFP only shows 104 mAh g^–1^, and capacity retention is improved from 88.9% in 800 °C-coated LFP to 94.0 % in 850 °C-coated LFP. If we reduce the amount of Fe_2_P phase by reducing coating time to 20 min, it results in a large improvement to the electrochemical performance with the sample delivering 125 mAh g^–1^ after 100 cycles with capacity retention of 100 %.

To clarify the influence of H_2_ gas, 60 nm LFP annealed in different atmospheric conditions were tested and shown in Fig. 4b. It can be seen that the 60 nm LFP annealed in Ar shows a stable cycle performance for 100 cycles without capacity decaying. On the contrary, the 60 nm LFP annealed in Ar/H_2_ shows a lower capacity of 140 mAh g^–1^ in the first cycle and drops to 127 mAh g^–1^ after 100 cycles, with a retention rate of 90.7%. In order to find the reason, electrochemical impedance spectra of 60 nm LFP and 560 nm LFP annealed in Ar and Ar/H_2_ were recorded before cycling (Supplementary Fig. 9 and Supplementary Table 1). It is shown that 60 nm LFP with addition of H_2_ gas, a increase of resistance is observed. The high resistance comes from the big size Fe_2_P rod formed in LFP sampled annealed in Ar/H_2_, whichblocks the transport of electrons/lithium ions (Fig. 3c). To sum up, these results indicate that carefully controlling Fe_2_P content can increase the capacity of half-cell LFP electrode by improving electronic conductivity and allowing for the formation of a conductive percolating network throughout the electrode (Fig. 4c)^20,29,30^. This improvement can also be attributed to the formation of a lithium pyrophosphate phase (Li_4_P_2_O_7_) during the carbon coating process, as shown in the Supplementary Fig. 10. Lithium phosphate is known to be a good lithium ion conductor and will aid in improving electrochemical performance^31^.

## Discussion

The formation of conductive Fe_2_P phase has been previously reported by Nazar et al^12^. However, only EDS information was presented that demonstrated an increased ratio of Fe/P at the grain boundary of LFP slices. In a later study published by Chung et al^32^, 3D phase morphology of Fe_2_P was determined by a combination of electron tomography using high-angle annular dark-field scanning transmission electron microscopy, EDS, and electron energy-loss spectroscopy. However, detailed study regarding phase formation mechanism and control of the secondary phase during carbon coating does not exist. In our work, we demonstrate the importance of carbon coating effect on the formation of Fe_2_P. Supplementary Figs. 11 and 12 show the surface phase evolution with carbon coating time, with chemical composition changing on LFP ingot, some spherical-like Fe-rich phase is formed on the surface.

**Fig. 5:**
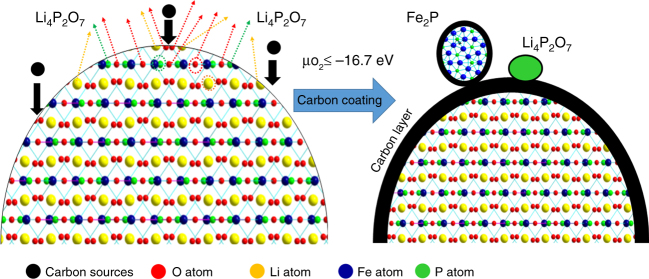
Oxygen partial chemical potential µO_2_. Schematic illustration of Fe_2_P phase formation during carbon coating process at reducing condition with regards to oxygen partial chemical potential

Formation of Fe_2_P phase is dependent on size, annealing temperature, and atmosphere, which is ascribed to the interface reaction between surface carbon and underlying LFP phase. Previously, our group proposed a carbon-competitive diffusion/deposition theory to explain the size-dependent phenomenon of impurity growth in LFP^23^. The formation of carbon layer on LFP involved a competition of carbon deposition on surface and carbon diffusion into bulk LFP. Smaller particles underwent a high rate of carbon deposition, resulting in rapid encapsulation of the LFP particle with carbon. This resulted in limited carbon diffusion to bulk LFP, leading to a thinner surface reduced layer. On the contrary, bigger particles consumed carbon slowly and had a slow carbon deposition rate, resulting in the carbon atoms being submerged into the crystalline lattice of LFP. Then, the surface of LFP was reduced to a secondary phase. Although this theory allowed us to postulate interface reactions occurring between carbon and LFP, it provides little insight toward the effect of temperature and reducing gas with carbon decomposition. Therefore, we provide a new direction in understanding Fe_2_P phase formation mechanism during carbon coating from a thermodynamic point of view with supporting evidence. (Fig. 5)

Based on the result shown above, Fe_2_P phase formation on LFP during carbon coating maybe expressed as follows:

1 $$2{\mathrm{C}}_{\mathrm{x}}{\mathrm{H}}_{\mathrm{y}} = 2{\mathrm{xC}} + {\mathrm{yH}}_2 \uparrow$$

2 $$4{\mathrm{LiFePO}}_4 + 9{\mathrm{C}} = 2{\mathrm{Fe}}_2{\mathrm{P}} + {\mathrm{Li}}_4{\mathrm{P}}_2{\mathrm{O}}_7 + 9{\mathrm{CO}} \uparrow$$

3 $$4{\mathrm{LiFePO}}_4 + 9{\mathrm{H}}_2 = 2{\mathrm{Fe}}_2{\mathrm{P}} + {\mathrm{Li}}_4{\mathrm{P}}_2{\mathrm{O}}_7 + 9{\mathrm{H}}_2{\mathrm{O}} \uparrow$$

The carbon coating process at high temperature involves decomposition of hydrocarbon sources into elemental carbon and reducing gas, accompanied by the formation of Fe_2_P and lithium phosphate at the LFP surface. At moderate temperatures (800 °C in LFP ingot in Supplementary Fig. 13 and 14), the amount of reducing gas produced is limited, no Fe_2_P is formed. As temperature increases to 875 °C, an increased amount of reducing gas is created and Fe_2_P formation occurs on the surface of LFP, as shown in Supplementary Fig. 13–15. Such a phenomenon is in agreement with first principle calculations performed by Ceder et al^33^. Their results suggest that Fe_2_P phase formation begins when the oxygen chemical potential $\mu _{o_2}$= –16.7 eV. $\mu _{o_2}$ is determined by temperature and oxygen partial pressure, simultaneously. Higher temperatures, lower oxygen partial pressures and/or the presence of reducing agents corresponds to lower values of $\mu _{o_2}$. It can explain the temperature dependent phenomenon of Fe_2_P phase during carbon coating. However, we find that the formation of Fe_2_P phase during carbon coating is dependent not only on temperature, but also on LFP particle sizes.

LFP particles with different sizes exhibiting different catalytic properties on the decomposition of hydrocarbon were reported in our previous paper. The study demonstrated that smaller LFP particles with large surface area facilitated carbon reduction reaction, resulting in an increased reactivity. The Brunauer–Emmett–Teller surface area of LFP particle was measured through N_2_ adsorption/desorption isotherms tests (Supplementary Fig. 16 and Supplementary Table 2), the results suggested that Fe_2_P formation was closely related to the surface area of LFP particles. In addition, carbon content of LFP particle was measured through TGA tests, and smaller LFP with higher carbon content was observed (Supplementary Fig. 17 and Supplementary Table 3). Thus, with increased surface area for 60 nm and 560 nm LFP, the amount of reducing gas produced are increased for smaller LFP particles, leading to a lower $\mu _{o_2}$ value compared to larger LFP particles undergoing the same process. As a result, the formation of Fe_2_P phase at 850 °C for 60 nm LFP and 560 nm LFP is easily attainable. However, for 19 µm LFP particle, a temperature of 900 °C is required for the formation of Fe_2_P phase, suggesting a size-related phenomenon. Based on Ceder's paper, such a Fe_2_P phase change phenomenon was easily observed in Li-rich LFP^33^. Our inductively coupled plasma atomic emission spectroscopy (ICP-AES) testing (Supplementary Table 4) on LFP particles also confirms that lithium content is higher in smaller LFP samples.

Furthermore, a reducing gas of Ar/H_2_ is intentionally introduced during the coating process to create a low oxygen partial pressure $\mu _{o_2}$. As expected, Fe_2_P phase formation occurs readily at a lower temperature of 700 °C for 60 nm LFP and 560 nm LFP, and 800 °C for 19 µm LFP in Ar/H_2_. To further support our observation, we applied the reducing atmosphere for LFP ingot in the carbon coating process. As shown in Supplementary Fig. 18, the spherical-like iron rich Fe_2_P phase is formed on LFP surface at 800 °C, indicating that Fe_2_P phase formation has been shifted to lower temperature by altering the annealing environment to a more reducing condition. Moreover, the Fe_2_P phase formation is independent of carbon precursor choice as Fe_2_P phase formation occurs in nano-LFP with gas carbon (C_2_H_4_) as precursor (Supplementary Fig. 19).

To conclude, we have shown that the formation of surface secondary phase in LFP during carbon coating is dependent on particle size, annealing temperature, and reducing atmosphere. The Fe_2_P phase transformation process is governed by thermodynamic rules and reaction kinetics. In a mild reducing atmosphere of argon gas, Fe_2_P phase is formed at high temperatures for all sizes of LFP, but the critical temperature for phase formation in small LFP is lower. Formation of Fe_2_P phase is closely related to reducing environment during the carbon coating process and is easily formed when the oxygen partial potential $\mu _{o_2}$ is decreased. Our preliminary data from half-cell show a positive effect on electrochemical performance of LFP with presence of Fe_2_P through fine-tuning the phase composition. However, the real impact of cycle life must be evaluated using long-term cycling in a range of temperatures with a full cell format. Nevertheless, method developed in this work may be extended to other olivine phosphate or insulated electrode that undergo carbon coating.

## Methods

### Carbon coating on LFP

The LFP ingot sample was provided by Phostech Lithium Inc. (Now, Johnson Matthey, Montreal, Canada), and the carbon coating process followed our previous report^23^.

Here, LFP ingot was used as a starting material and reduced to the appropriate size. An ingot sample was used for two main reasons: first one is its commercial availability providing us with a good baseline for understanding ordinary LFP materials. Second, an ingot offers a flat, smooth, and polished surface that is ideal for observing and investigating surface chemistry changes during carbon coating—for both the bulk olivine phase and secondary phases. The experimental details regarding melt-casting of LFP ingot can be found in our previous work^27,28^. In order to obtain a flat surface on the ingot, we polished the sample using sandpaper (London, 3 M Canada) ranging from coarse (Grit 120) to fine grade (Grit 1500). Following polishing, the samples were cleaned in an ultra-sonicator using ethanol several times.

LFP ingots were ball-milled into particle with different sizes using ZrO_2_ balls. Size distribution was controlled by ball milling for different times and using different sized ZrO_2_ balls. The ball milling process used isopropyl alcohol as the media, after the ball-mill process, the obtained precursors were dried in vacuum oven before carbon coating. The particle sizes of LFP were estimated with Image J software by calculating at least 5–10 SEM images.

The carbon-coating experiments on ingot samples were performed in a spray-pyrolysis system, which was previously developed for the synthesis of various nanomaterials. In the experiment, alcohol (anhydrous, chemical grade) was directly used as a carbon precursor and Ar as a carrier and protecting gas. Briefly, the ingot sample with the flat surface facing upward was placed in a quartz tube in Al_2_O_3_ crucibles with a tight seal using vacuums gears. Ar (80 sccm) (or with H_2_, 10 sccm), was introduced into the quartz tube for 20 min to eliminate any oxygen and to create an inert environment. Next, the furnace was heated at a heating rate of 5 °C min^–1^, and the carbon-coating process was performed at temperatures ranging from 800 °C to 900 °C for 20 min. After the carbon coating process at high temperature, the coated sample was cooled to room temperature naturally within the furnace still under the protection of the Ar atmosphere. Samples were collected for further experiments once they were cooled down.

Carbon coating of LFP powders were similar to ingot samples, except that we used the lactose as the carbon sources instead of alcohol. The LFP powders were fully mixed with lactose using water or isopropyl alcohol (carbon source content was 10 wt. %, the concentration was 2 g L^–1^ based on weight of LFP), then the suspension was mixed ultrasonically, and was then allowed to evaporate to dryness. The sample was annealed in Ar (with H_2_) atmosphere at a ramp rate of 5 °C min^–1^, from 600 °C to 900 °C.

### Physical characterization

Crystal structure and phase composition of carbon-coated LFP were collected using the X-ray diffraction (D8 Advance, Bruker) in the range of 10–90° with a step of 0.01° per seconds. Slow scans in the range of 28–33° were also performed to provide detail on the formation of impurity phases. The carbon-coated LFP powders were subjected to Hitachi 4800 SEM equipped with EDS detector. The working voltage employed for EDS mapping was 20 kV. The Raman spectra were conducted at HORIBA Scientific LabRAM with a laser (*λ* = 532.3 nm) as the excitation source. Raman spectroscopy maps from at the sample were collected in autofocus mode with a spatial resolution of ca. 2 µm. The detailed structure of carbon-coated LFP particles are investigated by HR-TEM (JEOL 2010 FEG) operating at an accelerating voltage of 20 kV, diffraction patterns were recorded using the SAED mode. Thermo-gravimetric analysis (TGA) was carried out on a TA SDT Q600 in an air atmosphere from room temperature to 900 °C at a rate of 10 C min^–1^. N_2_ adsorption/desorption isotherms of LFP particles were performed using a Folio Micromeritics TriStar II Surface Area and Pore Size Analyzer.

### Electrochemical tests

The electrochemical performance of carbon-coated LFP were tested in 2032 coin cells, using a Li metal foil as a counter electrode. The electrode is composed of 80 wt.% carbon-coated LFP as active material, 10 wt.% PvDF as binder, and 10 wt.% carbon black as conductive agent, with an active material loading of 1.5–2 mg cm^–2^. 1 M LiPF_6_ in EC, DEC, and EMC with a volume ratio of 1:1:1 was employed as an electrolyte along with Celgard 2400 as a separator. The cells were assembled in an Ar-filled glove box with oxygen and water levels below 1 ppm. Charge–discharge cycling using a constant current mode was performed on an Arbin BT-2000 Battery Test System. All the electrochemical measurements were carried out in a voltage range of 2.2–4.2 V at RT.

### Data availability

The data that support the findings of this study are available from the corresponding author upon request.

## Electronic supplementary material

Supplementary Information

## References

[1] Padhi AK, Nanjundaswamy KS, Goodenough JB (1997). Phospho olivines as positive electrode materials for rechargeable lithium batteries. J. Electrochem. Soc..

[2] Wang J, Sun X (2015). Olivine LiFePO_4_: the remaining challenges for future energy storage. Energy Environ. Sci..

[3] Wang Y, He P, Zhou H (2011). Olivine LiFePO_4_: development and future. Energy Environ. Sci..

[4] Wang J, Sun X (2012). Understanding and recent development of carbon coating on LiFePO_4_ cathode materials for lithium-ion batteries. Energy Environ. Sci..

[5] Herstedt M (2003). Surface chemistry of carbon-treated LiFePO_4_ particles for Li-ion battery cathodes studied by PES. Electrochem. Solid State Lett..

[6] Wilcox JD, Doeff MM, Marcinek M, Kostecki R (2007). Factors influencing the quality of carbon coatings on LiFePO_4_. J. Electrochem. Soc..

[7] Chen L, Yuan YQ, Feng X, Li MW (2012). Enhanced electrochemical properties of LiFe_1−x_Mn_x_PO_4_/C composites synthesized from FePO_4_·2H_2_O nanocrystallites. J. Power Sources.

[8] Oh SW (2010). Double carbon coating of LiFePO_4_ as high rate electrode for rechargeable lithium batteries. Adv. Mater..

[9] Wang J (2013). Interaction of carbon coating on LiFePO_4_: A local visualization study of the influence of impurity phases. Adv. Funct. Mater..

[10] Yu DYW (2008). Impurities in LiFePO_4_ and their influence on material characteristics. J. Electrochem. Soc..

[11] Trudeau ML (2011). In situ high-resolution transmission electron microscopy synthesis observation of nanostructured carbon coated LiFePO_4_. J. Power Sources.

[12] Herle PS, Ellis B, Coombs N, Nazar LF (2004). Nano-network electronic conduction in iron and nickel olivine phosphates. Nat. Mater..

[13] Kang B, Ceder G (2009). Battery materials for ultrafast charging and discharging. Nature.

[14] Wagemaker M, Ellis BL, Lutzenkirchen-Hecht D, Mulder FM, Nazar LF (2008). Proof of supervalent doping in olivine LiFePO_4_. Chem. Mater..

[15] Chung SY, Choi SY, Kim TH, Lee S (2015). Surface-orientation-dependent distribution of subsurface cation-exchange defects in olivine-phosphate nanocrystals. ACS Nano.

[16] Paolella A (2015). Cation exchange mediated elimination of the Fe-antisites in the hydrothermal synthesis of LiFePO_4_. Nano Energy.

[17] Paolella A (2016). Accelerated removal of Fe-antisite defects while nanosizing hydrothermal LiFePO_4_ with Ca^2+^. Nano. Lett..

[18] Xu Y, Lu Y, Yan L, Yang Z, Yang R (2006). Synthesis and effect of forming Fe_2_P phase on the physics and electrochemical properties of LiFePO_4_/C materials. J. Power Sources.

[19] Lin Y, Gao MX, Zhu D, Liu YF, Pan HG (2008). Effects of carbon coating and iron phosphides on the electrochemical properties of LiFePO_4_/C. J. Power Sources.

[20] Song MS (2008). Amphoteric effects of Fe_2_P on electrochemical performance of lithium iron phosphate–carbon composite synthesized by ball-milling and microwave heating. J. Power Sources.

[21] Yin Y (2012). High-rate capability of LiFePO_4_ cathode materials containing Fe_2_P and trace carbon. J. Power Sources.

[22] Zhang L (2015). Synthesis of Fe_2_P coated LiFePO_4_ nanorods with enhanced Li-storage performance. J. Alloy. Compd..

[23] Wang J (2014). Size-dependent surface phase change of lithium iron phosphate during carbon coating. Nat. Commun..

[24] Zaghib K, Mauger A, Gendron F, Julien C (2007). Surface effects on the physical and electrochemical properties of thin LiFePO_4_ particles. Chem. Mater..

[25] Julien CM, Mauger A, Zaghib K (2011). Surface effects on electrochemical properties of nano-sized LiFePO_4_. J. Chem. Mater..

[26] Kayyar A, Qian H, Luo J (2009). Surface adsorption and disordering in LiFePO_4_ based battery cathodes. Appl. Phys. Lett..

[27] Gauthier M (2010). Melt casting LiFePO_4_ I. Synthesis and characterization. J. Electrochem. Soc..

[28] MacNeil D (2010). Melt Casting LiFePO_4_ II. particle size reduction and electrochemical evaluation. J. Electrochem. Soc..

[29] Liu Y, Cao C, Li J, Xu X (2010). A novel synthesis of Fe_2_P–LiFePO_4_ composites for Li-ion batteries. J. Appl. Electrochem.

[30] Kang HC (2008). Optimized solid-state synthesis of LiFePO_4_ cathode materials using ball-milling. J. Power Sources.

[31] Rho YH, Nazar LF, Perry L, Ryan D (2007). Surface chemistry of LiFePO_4_ studied by Mössbauer and X-ray photoelectron spectroscopy and its effect on electrochemical properties. J. Electrochem. Soc..

[32] Chung SY, Kim YM, Choi SY (2010). Direct physical imaging and chemical probing of LiFePO4 for Lithium-Ion batteries. Adv. Funct. Mater..

[33] Ong SP, Wang L, Kang B, Ceder G (2008). Li-Fe-P-O_2_ phase diagram from first principles calculations. Chem. Mater..

