# Supplementary material for: Formation of size-dependent and conductive phase on lithium iron phosphate during carbon coating
Source: Nat Commun. 2018 Mar 2;9:929. doi: 10.1038/s41467-018-03324-7 (PMC5834541; doi:10.1038/s41467-018-03324-7)
Supplement: Supplementary file 1 — Supplementary Information [file 41467_2018_3324_MOESM1_ESM.pdf]

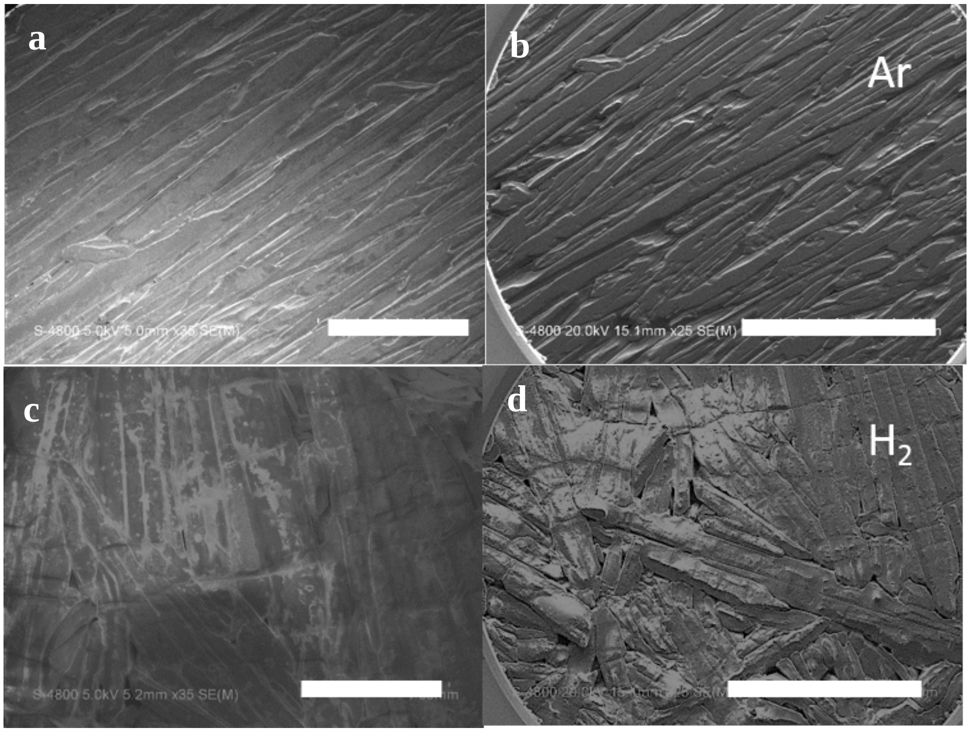

Supplementary Figure 1 |Surface phase on LFP Ingot in Ar and H_2. (a–d) SEM and BSE images of LFP after 900 °C. Scale bar 1 mm. We can observe flat surface is presented, no spherical phase could be found in inert Ar gas atmosphere and reducing H_2 atmosphere. Scale bar is 1 mm in (a, c), 2mm in (b, d)

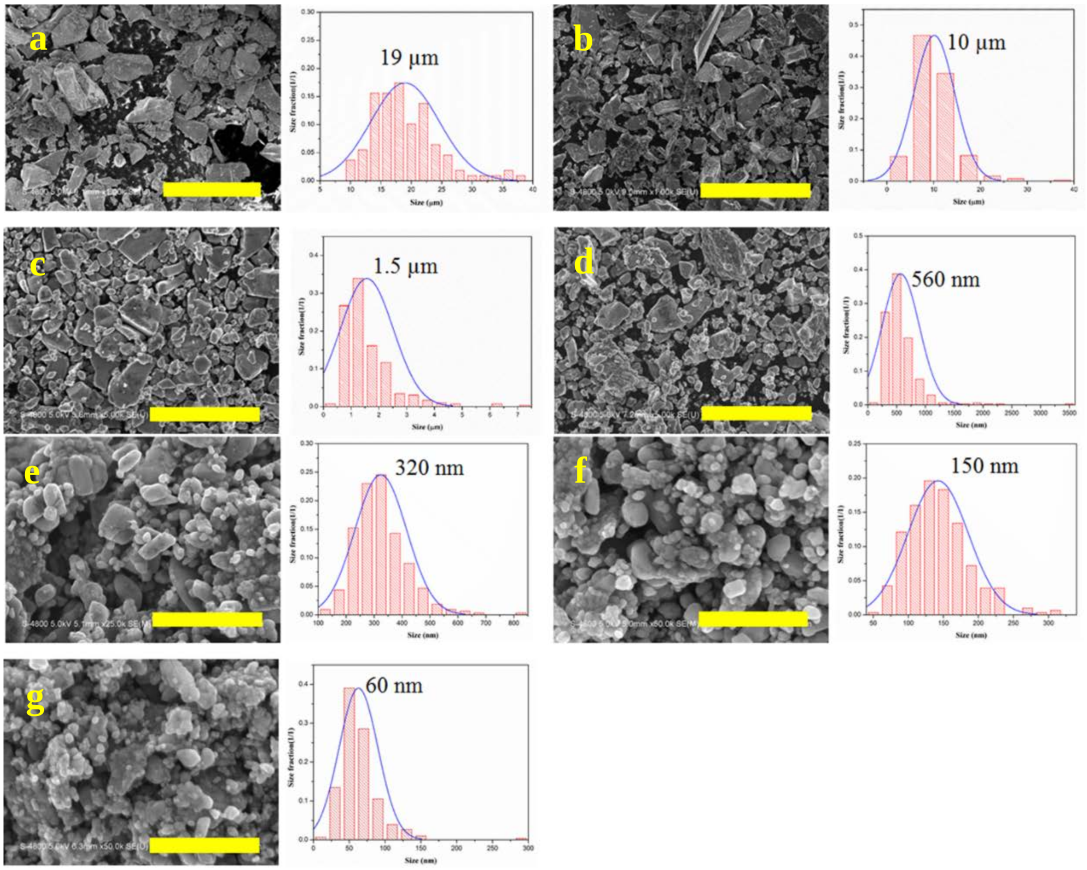

Supplementary Figure 2 |Size-distribution of ball-milled LFP particles. (a–g) FE-SEM images and the size distribution profiles of different sizes. Scale bar is 50 μm in (a, b), 10 μm in the (c, d), 2 μm (e–f), 1 μm (g).

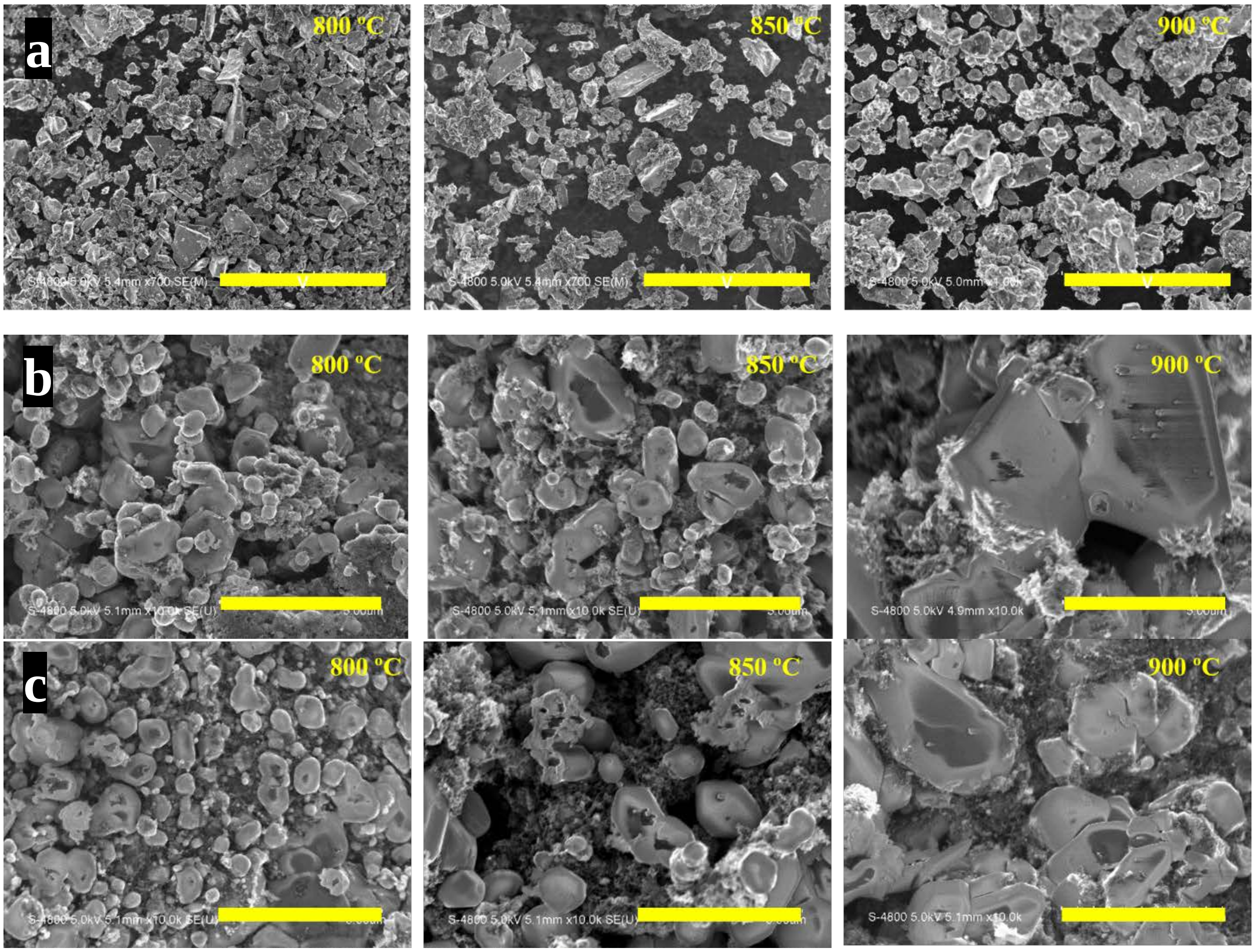

Supplementary Figure 3 |Surface change of LFP particles at high temperature. Morphology changes of different size LFP after high temperature annealing in Ar. (a) 19 µm, (b) 560 nm, (c) 60 nm. Scale bar, 50 µm in (a) and 5 µm in (b, c).

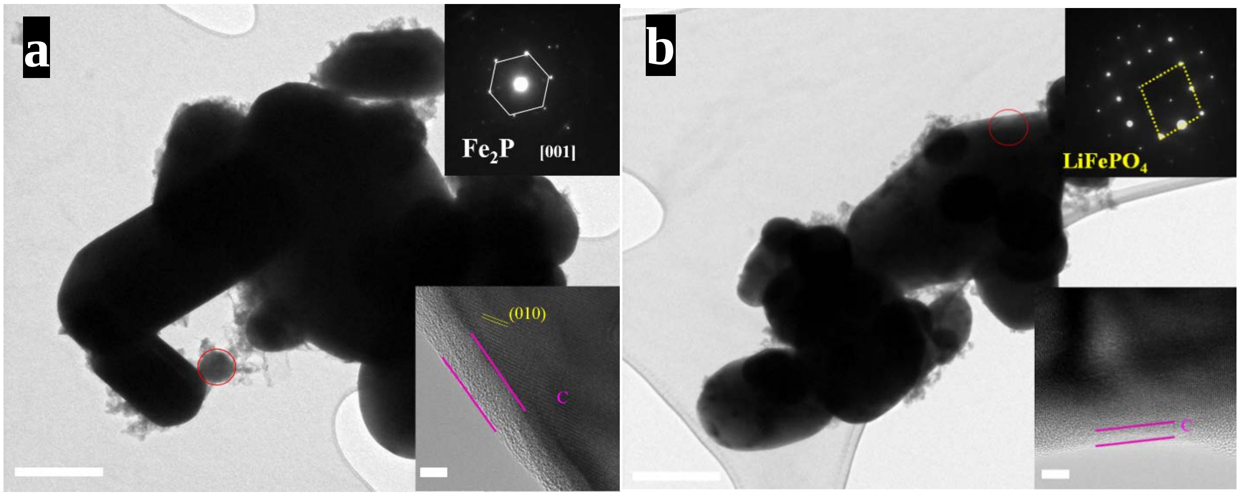

Supplementary Figure 4 |Phase composition of 560 nm LFP after carbon coating. HRTEM of 560 nm LFP after high temperature annealing in Ar at 900 °C, with Fe_2P phase (a) and LFP phase mixed (b). Scale bar, 500 nm in TEM, 5 nm in HRTEM

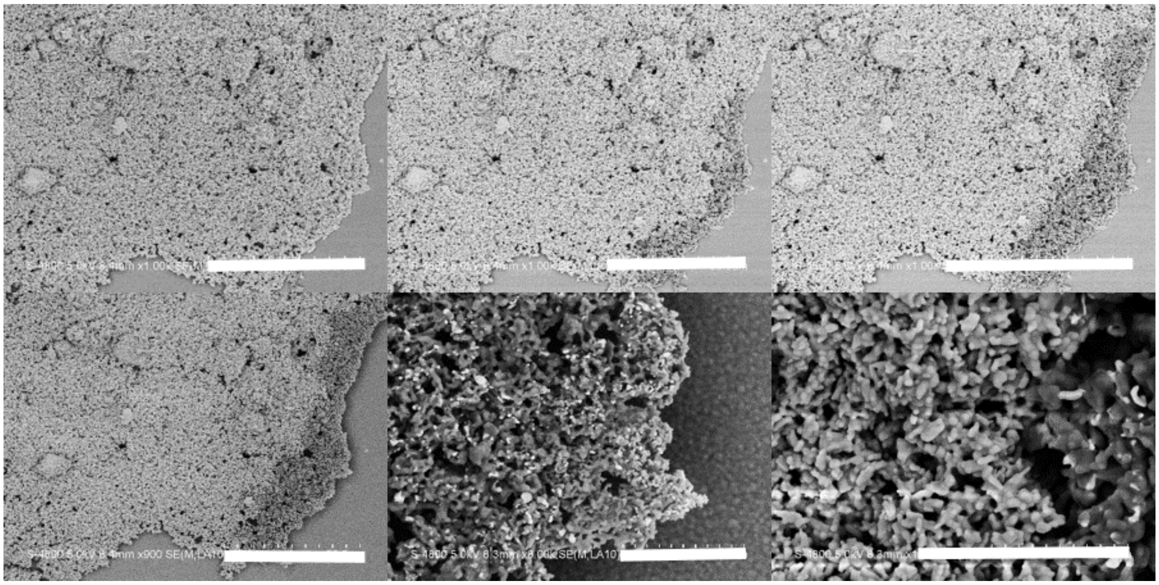

Supplementary Figure 5 |In-situ carbon coating experiment. SEM observation of nano-size LFP particles. Scale bar is 50 µm.

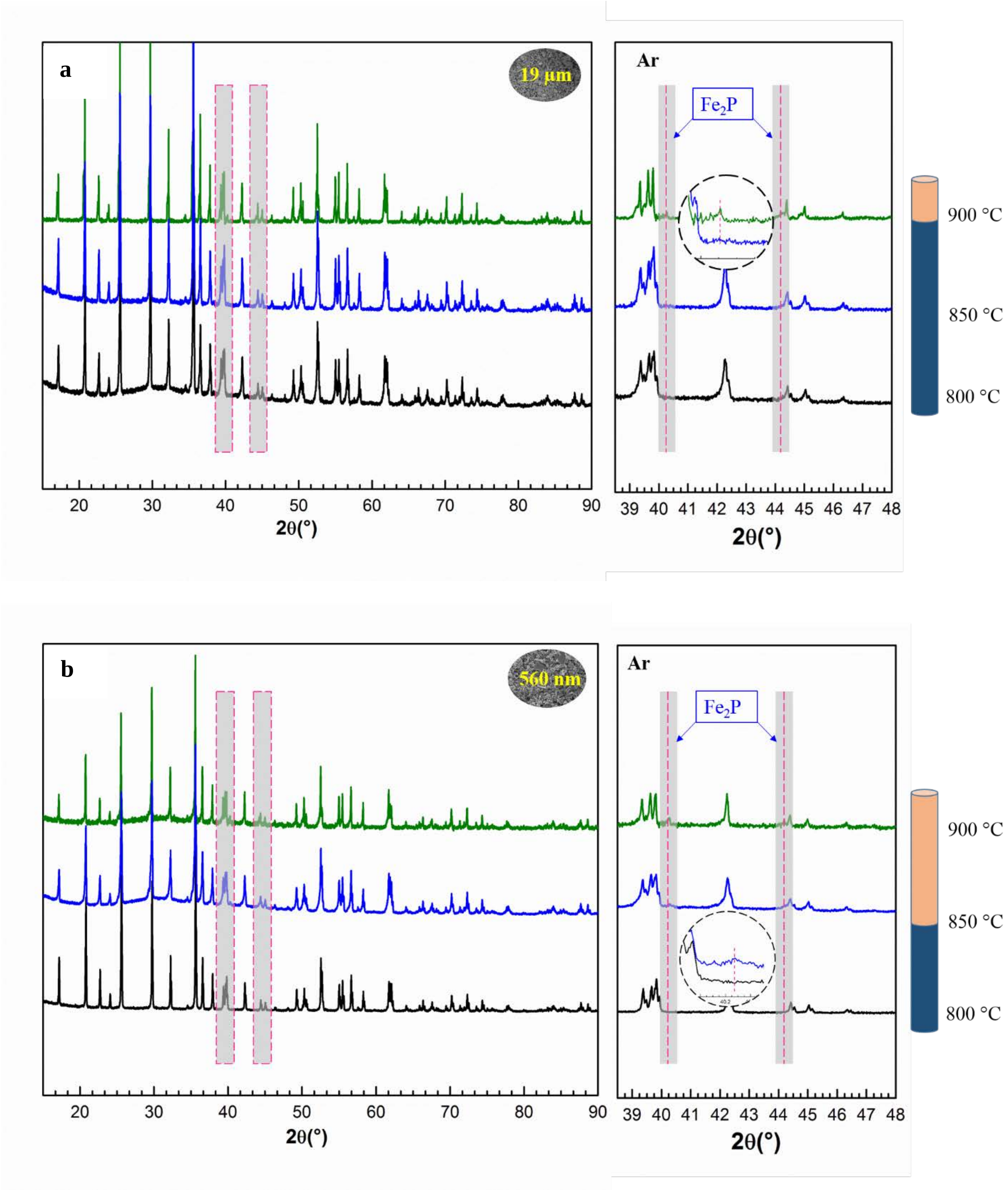

Supplementary Figure 6 |Size and temperature dependent phenomenon in Ar. Temperature dependant properties of Fe_2P phase formation after carbon coating in Ar (a) for 19 micron LFP and 560 nm LFP (b) particles. The Fe_2P phase formation temperature is 900 ^{o}C and 850 ^{o}C, respectively.

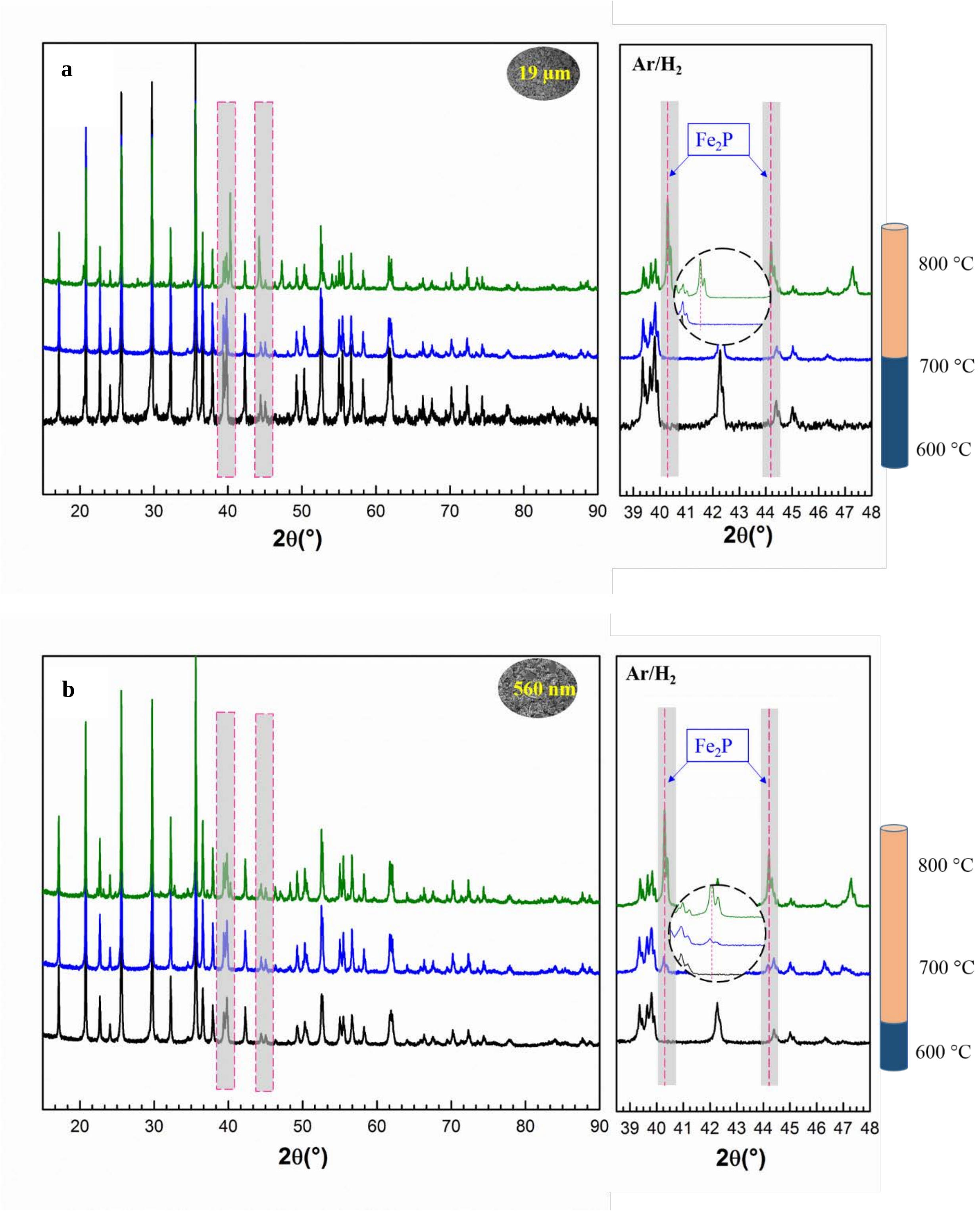

Supplementary Figure 7 |Size and temperature dependent phenomenon in Ar/H_2. Temperature dependant properties of Fe_2P phase formation after carbon coating in reducing atmosphere (a) for 19 micron LFP and (b) 560 nm LFP. The Fe_2P phase formation temperature is 800 °C and 700 °C, respectively.

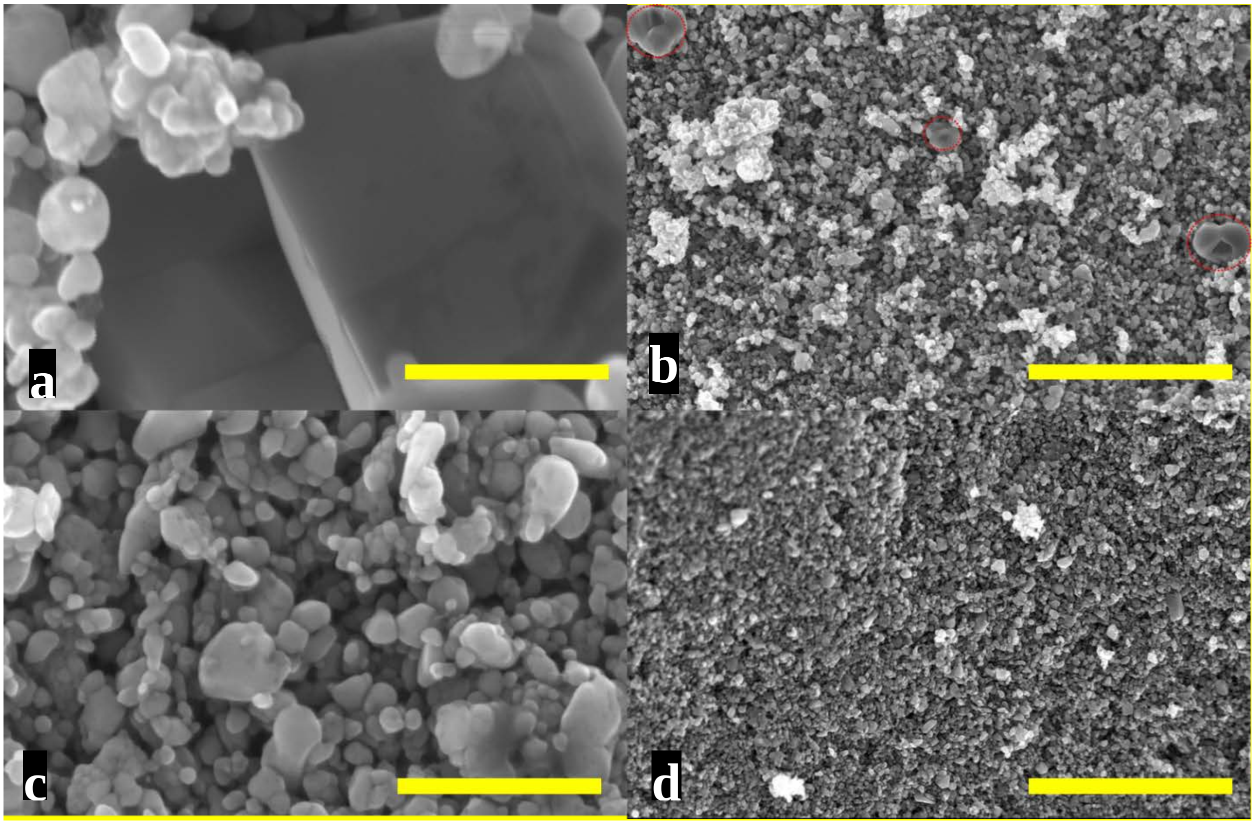

Supplementary Figure 8 |Atmospheric dependent phenomenon of 60 nm LFP. Morphology changes of LFP after high temperature annealing at 700 C. (a, b) 60 nm in Ar/H2 gas, (b,d) 60 nm LFP in Ar gas. Scale bar, 200 nm in (a,c) and 3 μm in (b, d). It is seen that there are some big crystals formed in LFP after carbon coating in reducing atmosphere.

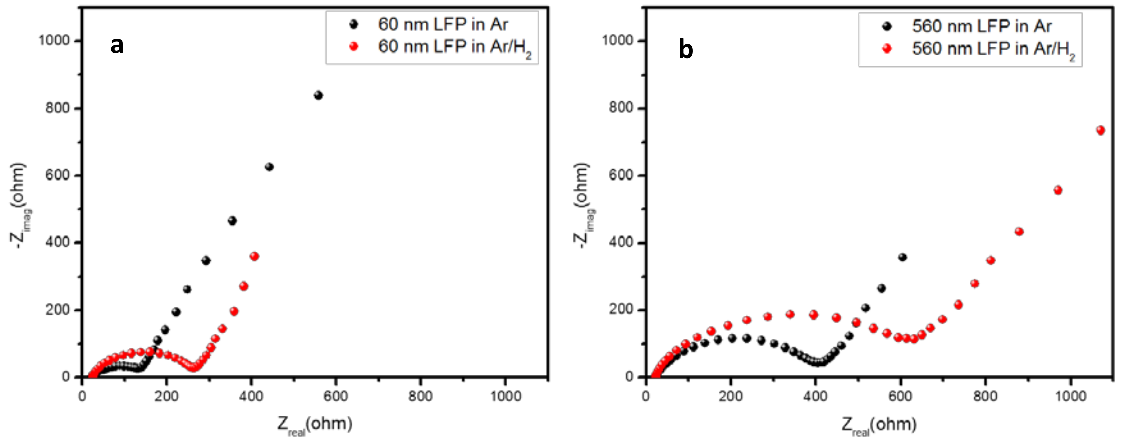

Supplementary Figure 9 |Electrochemical resistance measurements. Electrochemical impedance spectra of 60 nm LFP (a) and 560 nm LFP (b) annealed in different atmosphere.

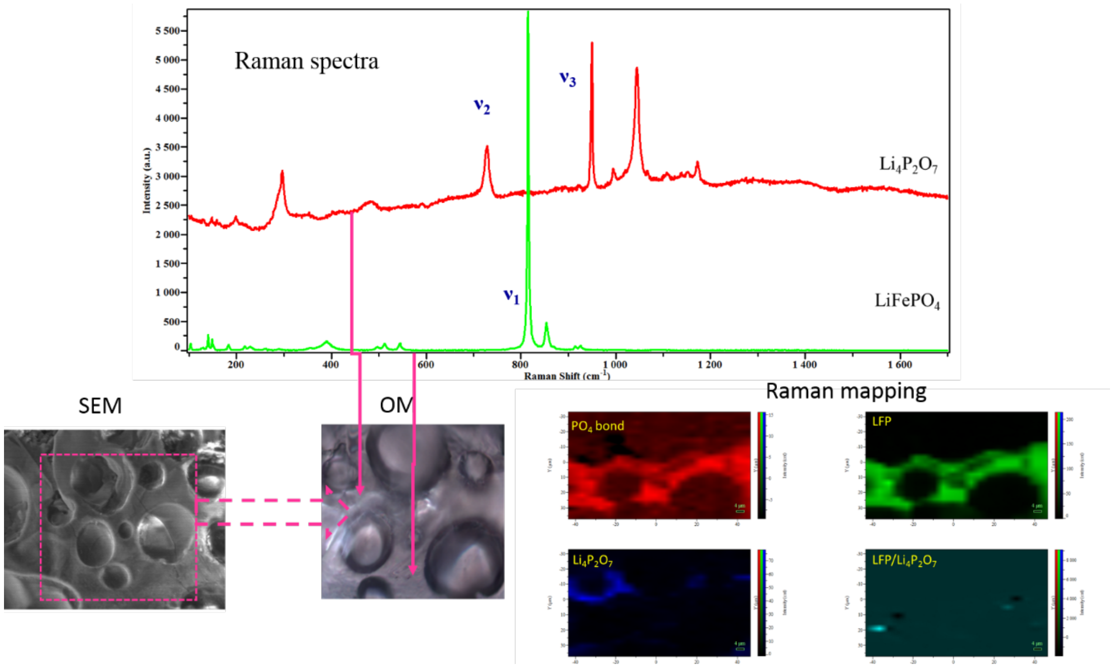

Supplementary Figure 10 |SEM and Optical images of Ingot surface. After carbon coating on LFP surface, it is seen that lithium pyrophosphate is formed at the vicinity of spherical like phase from the Raman spectra and mapping. Peak ν_1 (953 cm^{-1}) is related to the symmetric stretching bond of P-O, which is one of characteristic peak of LFP material based on standard sample. Two peaks of asymmetric bend bond (ν_2, 731 cm^{-1}) and asymmetric stretch bond (ν_3, 1046 cm^{-1}) are the characteristic peaks for $Li_4P_2O_7$ phase based on the standard sample.

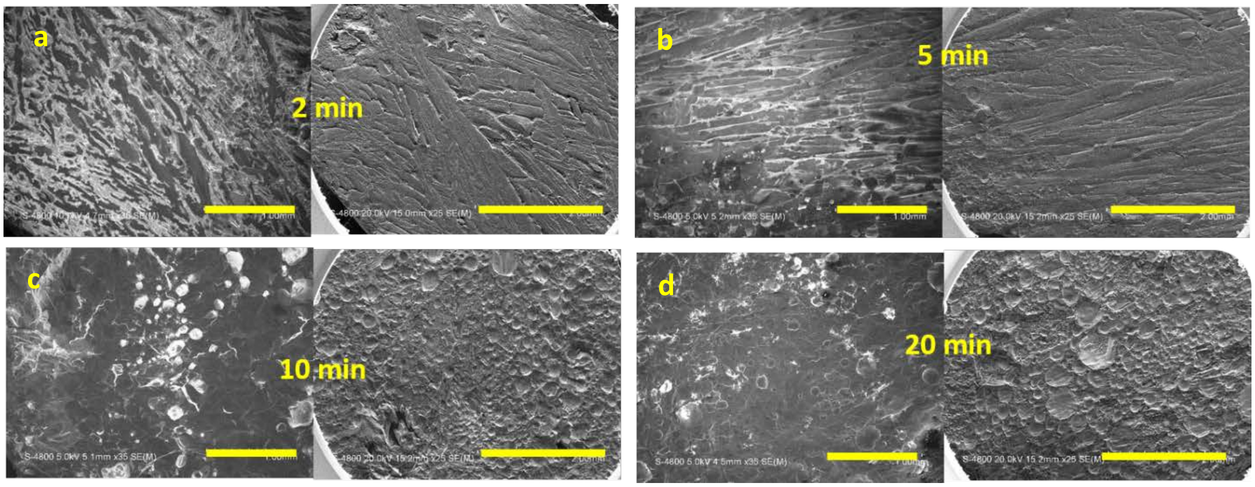

Supplementary Figure 11 |SEM and BSE images of Ingot surface. At 2 min, ingot surface is flat, no change can be observed. When the time increase to 5 min, some smaller ball shaped phase is observed at the edge of ingot. At 10 min, the whole ingot surface is composed of many ball shaped islands, the ball has larger particles and small particles. If we further increase the time to 20 min, the ball shaped island becomes more obvious. Scale bar, 1mm in left image and 2 mm in right image for all pictures.

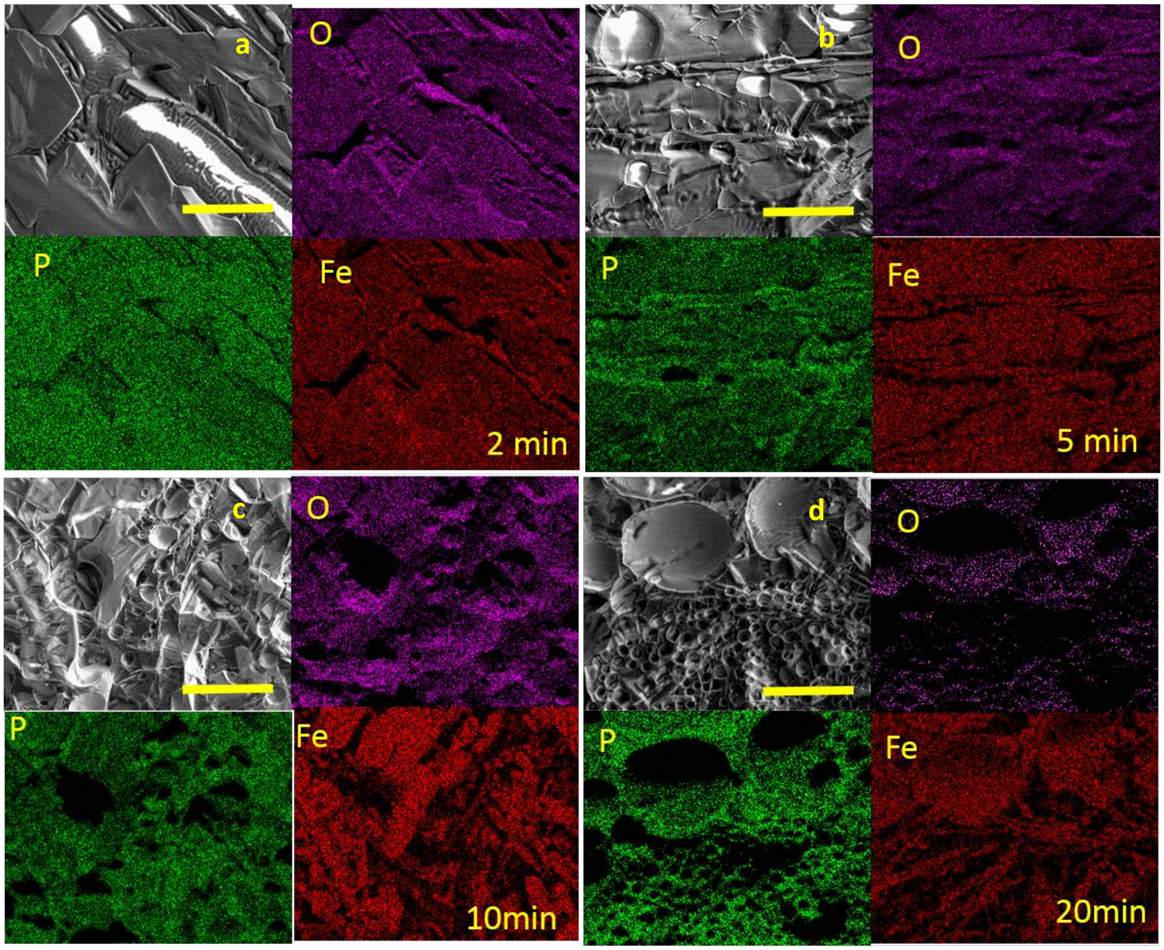

Supplementary Figure 12 |Mapping images of Ingot surface. More details is revealed by the mapping images. In the 2 min sample, we cannot see the existence of P deficient phase. In 5 min sample, the smaller P deficient phase is observed and confirmed by EDS mapping. In 10 min sample, the phase is becoming bigger and some smaller P deficient phase are observed. At longer time, the phase continue growing bigger and some smaller P deficient phase is observed. Scale bar is 200 µm.

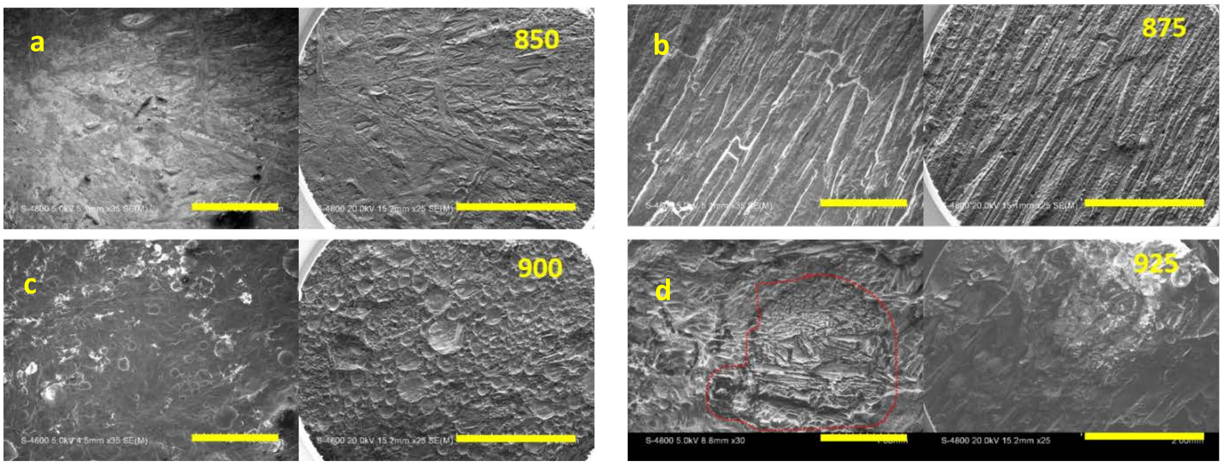

Supplementary Figure 13 |Surface phase on LFP Ingot. SEM and BSE images of LFP after carbon coating at 850 °C-925 °C with step of 25 °C. At 850 °C, we can observe flat surface which is covered by one layer of carbon. Increase the temperature to 875 °C, some smaller ball shaped phase is observed. At 900 °C, this phenomenon is more obvious and the whole ingot surface is composed of many ball shaped islands. If we further increase the temperature, the surface is melting down, but ball shaped island still can be observed on higher magnification SEM images. Scale bar, 1mm in left image and 2 mm in right image for all pictures.

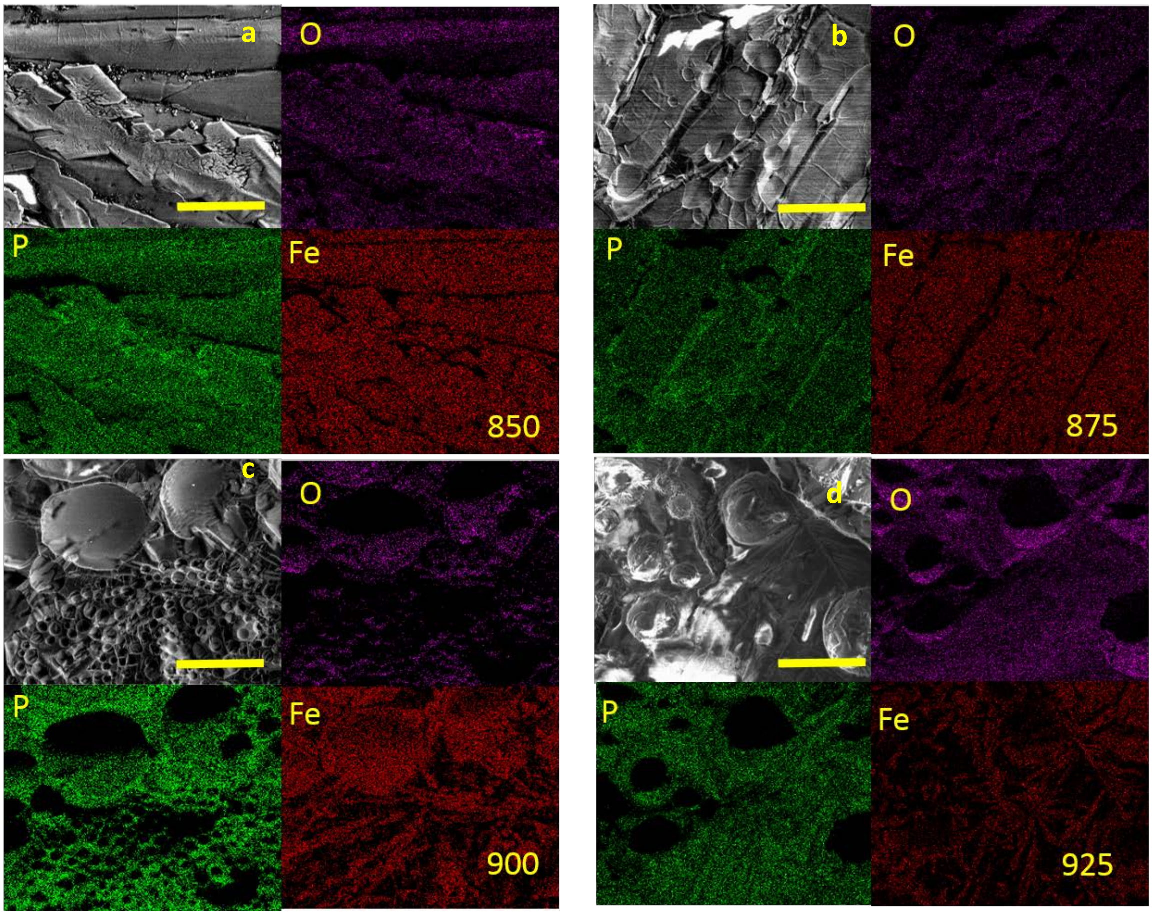

Supplementary Figure 14 |EDS mapping images of Ingot surface. More details is revealed by the mapping images. In the 875 °C sample, we can roughly see the existence of P deficient phase. In 900 °C sample, the phase is becoming bigger and a lot of smaller P deficient phase are also observed. At higher temperature, the phase continue growing bigger and become more deficient. Scale bar is 200 µm.

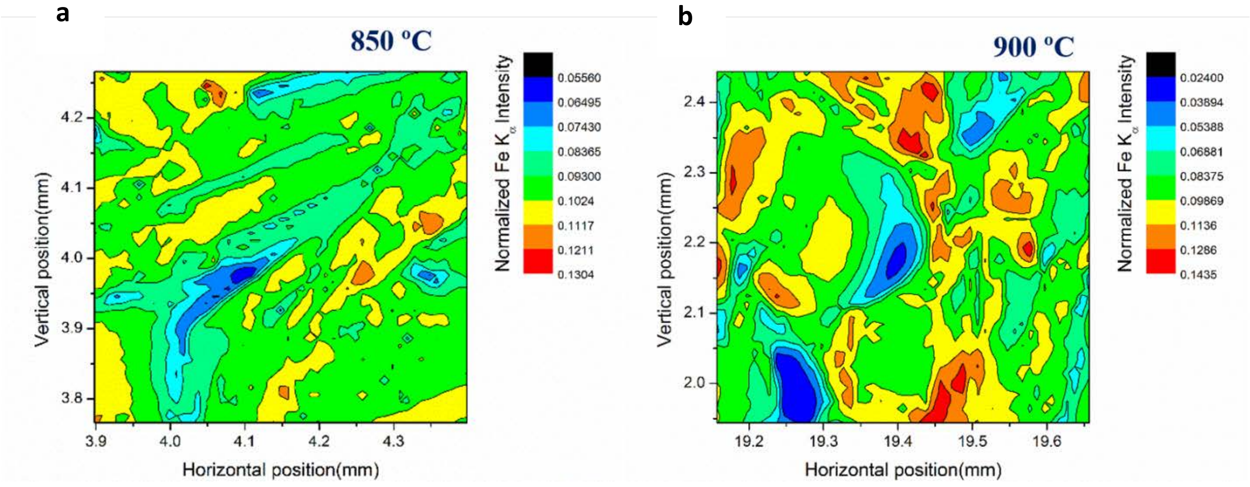

Supplementary Figure 15 |XRF Mapping images of Ingot surface. In the 850 °C sample, we can roughly see the existence of Fe rich phase. In 900 °C sample, the Fe rich phase in spherical shape are observed.

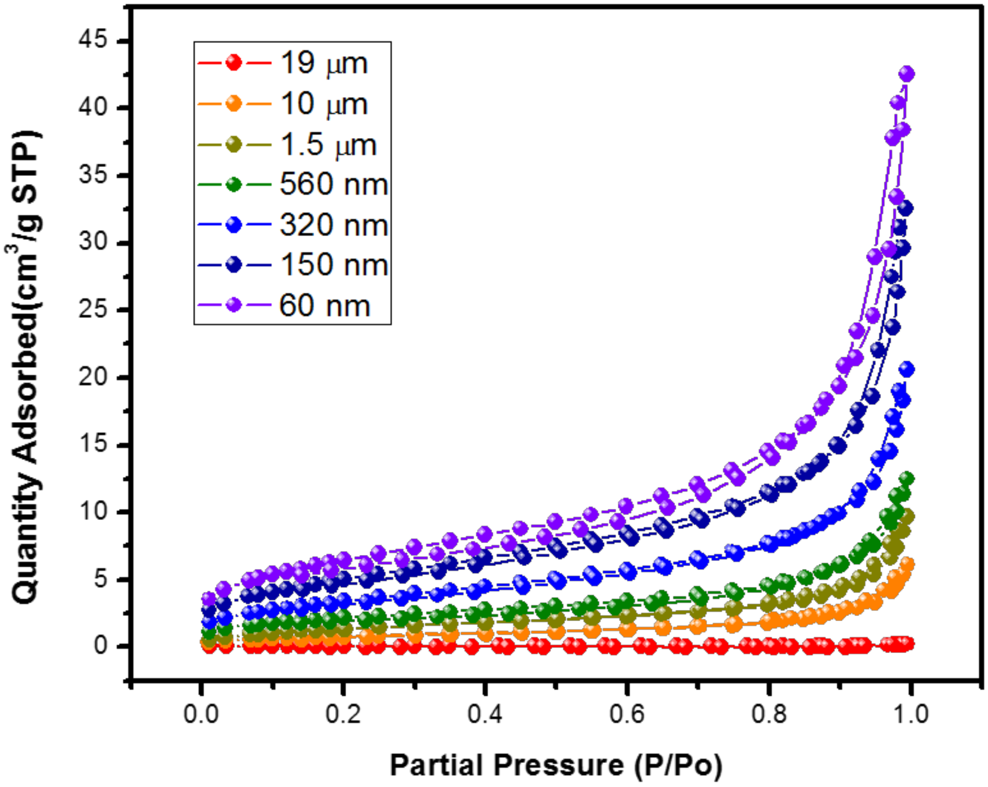

Supplementary Figure 16 |Surface area measurements. Nitrogen adsorption/desorption isotherms of different size LFP.

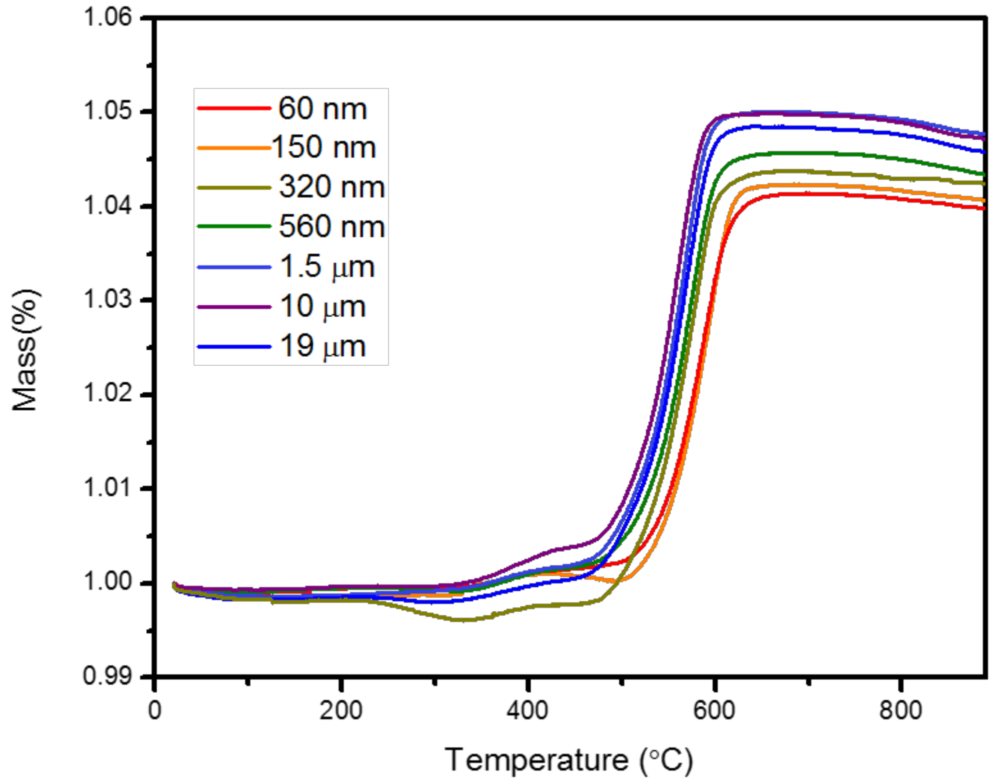

Supplementary Figure 17 |Carbon contents measurements. TGA curves of different size LFP after carbon coating at 900 °C.

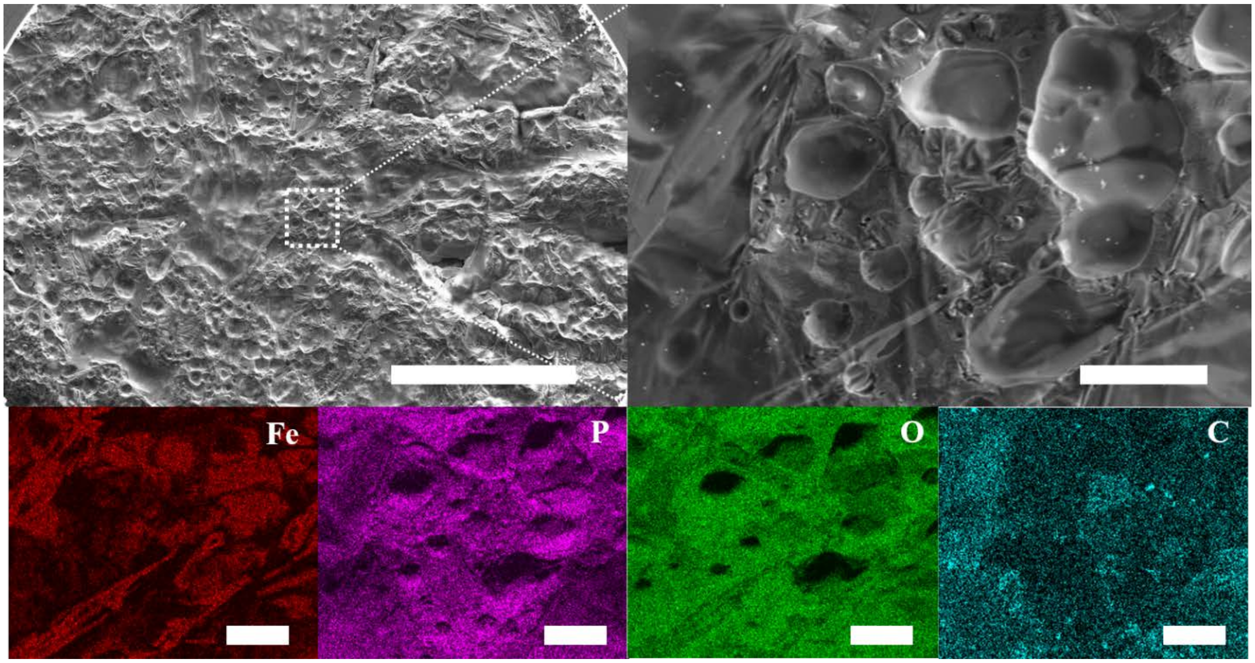

Supplementary Figure 18 |Surface conductive phase formation in Ar/H_2 at 800 ºC. (a, b) BSE images (c-f) and EDS mapping of surface conductive phase formation on $LiFePO_4$ after carbon coating. Scale bar is 200 µm.

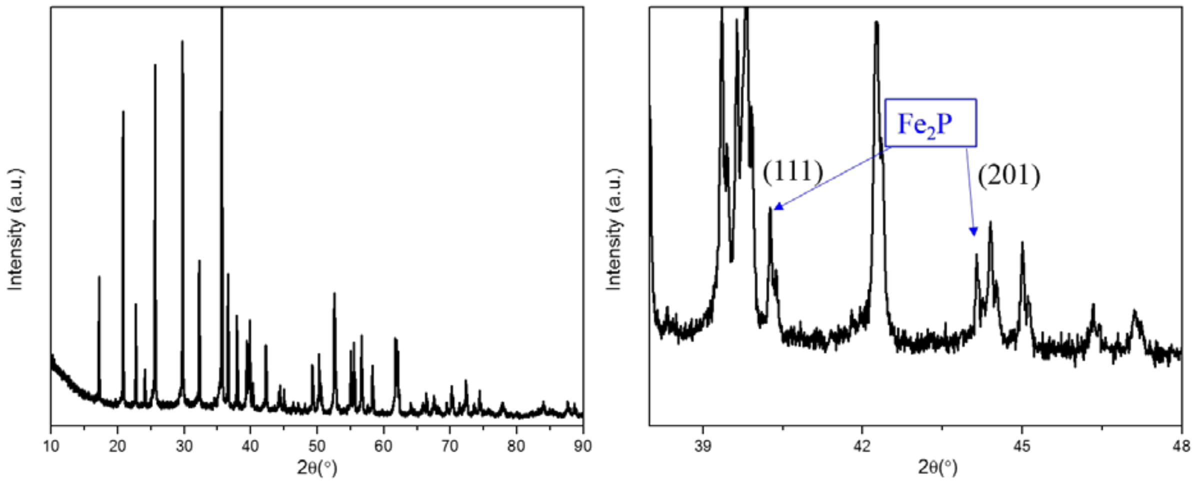

Supplementary Figure 19 |Carbon source influence. XRD pattern of 60 nm LFP using C_2H_4 as carbon sources.

Supplementary Table 1 |Electrochemical impedance summary. Electrode resistance of LFP electrodes

Sample	Re
**60 nm LFP in Ar**	133
**60 nm LFP in Ar/H2**	262
**560 nm LFP in Ar**	400
**560 nm LFP in Ar/H2**	631

Supplementary Table 2 |Surface area summary. BET surface areas of different size LFP particles

**Sample**	BET Surface area(m^2/g)
**60 nm**	23.5964
**150 nm**	18.4034
**320 nm**	12.5343
**560 nm**	7.9038
**1.5 µm**	5.2376
**10 µm**	2.9041
**19 µm**	0.2393

Supplementary Table 3 |Carbon content summary. Mass change and carbon contents of different size LFP after carbon coating at 900 °C

Sample	Mass change (wt.%)	Carbon content (wt.%)
60 nm	3.97	1.1
150 nm	4.06	1.01
320 nm	4.28	0.79
560 nm	4.35	0.72
1.5 µm	4.57	0.5
10 µm	4.72	0.35
19 µm	4.77	0.30

Supplementary Table 4 |Element composition summary. Element composition obtained by means of inductively coupled plasma atomic emission spectroscopy (ICP-AES) analyses for initial $LiFePO_4$ samples.

**Sample**	**Li (wt.%)**	**Fe (wt.%)**	**P (wt.%)**	**Li: Fe: P (molar ratio)**
19 um LFP	33.87	3.96	18.76	(1.001: 0.942: 1)
560 nm LFP	30.87	3.77	17.13	(0.999: 0.982: 1)
150 nm LFP	30.21	3.72	16.74	(1.000: 0.994: 1)

Supplementary Note 1 |The definition of oxygen chemcial potential

The oxygen chemical potential term, which can be written as:

$$\mu_{o_2}(T, P_{O_2}) = \mu_{o_2}(T, P_0) + kTln\frac{P_{O_2}}{P_0} \qquad \text{(Supplementary 1)}$$

where P_{O_2} is the partial pressure of oxygen, P_0 is a reference oxygen partial pressure, which is 0.1 MPa in this work, $\mu_{o_2}(T, P_0)$ is the oxygen chemical potential at the reference partial pressure and temperature T, and k is the Boltzmann's constant. From above equation, we know that μ_{o_2} is determined by the temperature and oxygen partial pressure. Higher temperature and strong reducing atmosphere result in smaller μ_{o_2} value.
